# Complete mitochondrial genome and phylogenetic position of the gall aphid *Chaetogeoica ulmidrupa* (Hemiptera: Aphididae)

**DOI:** 10.1080/23802359.2026.2638669

**Published:** 2026-03-05

**Authors:** Xiaonan Wang, Jiaqi Wu, Takahiro Yonezawa, Zhumei Ren

**Affiliations:** a School of Life Science, Shanxi University, Taiyuan, China; b Graduate School of Integrated Sciences for Life, Hiroshima University, Hiroshima, Japan

**Keywords:** Aphididae, *Chaetogeoica ulmidrupa*, gall aphid, mitochondrial genome, phylogeny

## Abstract

We sequenced two complete mitochondrial genomes of *Chaetogeoica ulmidrupa*, a gall-forming aphid on *Pistacia chinensis*. Each genome consists of 13 protein-coding genes, 22 tRNA genes, two rRNA genes and a control region, with a strong A+T bias (∼85%). All protein-coding genes initiate with ATN codon and terminate with TAA, except for ND4 and COX1 ending with a single T. Phylogenetic analysis strongly supported that *C. ulmidrupa* was sister to *C. yunlongensis* and together they formed a clade with *Slavum lentiscoides*, all three species of which feed on the primary host plant *P. chinensis* to form galls.

## Introduction

The gall aphid *Chaetogeoica ulmidrupa* Zhang (Hemiptera: Aphididae), a Fordini species specifically feeding on *Pistacia chinensis* Bunge (1835), was originally described as new from the aphid specimens stored at the Museum of the Institute of Zoology, Chinese Academy of Sciences (Zhang and Qiao 1998). This species later was cited in the review article on the diversity and host specificity of gall aphids in China (Chen and Qiao 2012). However, no further updates were published on this species to date. Seven species were identified in the genus *Chaetogeoica* (Aphididae: Fordini) (Favret 2025), and they all choose the *Pistacia* species as the primary host plant. In recent years, mitogenome has been employed as a powerful tool to analysis the molecular phylogenetics and population genetics (Kim et al. 2024; Wang et al. 2024). To date, only one mitochondrial genome in this genus, leaving the phylogenetic position and genetic distinctness of other members, like *C. ulmidrupa*, unresolved (Ren et al. 2017). The mitochondrial genes of *C. yunlongensis* along with those of other Aphididae taxa were used to reconstruct the phylogeny (Liang et al. 2024). In addition, one chromosome-level genome for *C. ovagalla* was assembled with a size of 289 Mb, containing 14,492 genes (Xu et al. 2024). As gall aphids, they are highly limited in molecular analyses, yet crucial to the study of the genetics, evolution and potential implications for the coevolutionary model with its host plant.

In this study, we sequenced two complete mitochondrial genomes of *C. ulmidrupa* and analyzed its phylogenetic position. This study provides valuable genetic data and resources information for further research on aphid phylogeny.

## Materials and methods

**Figure 1.**
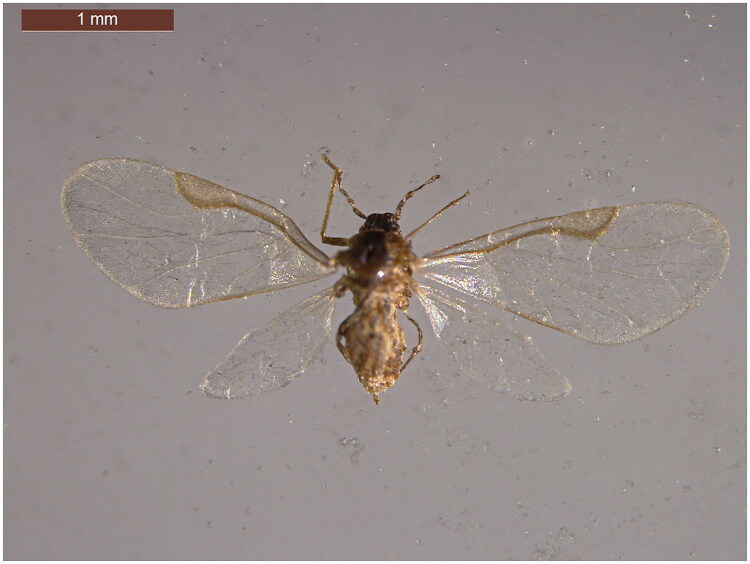
Reference image of *Chaetogeoica ulmidrupa* with sample ren_4512 as a representative (the picture taken by Yukang Liang in 2016).

We collected two individuals of *Chaetogeocia ulmidrupa* with the vouchers Ren_A1094 (Shiyan, Hubei, China; 32.63 N, 110.79E; July 2014) and Ren_A4512 (Baoji, Shaanxi, China; 34.36 N, 107.23E; June 2016). The aphid sample (sample Ren_A4512 as representative) is shown in Figure 1. All samples are stored at School of Life Science, Shanxi University (contact Zhumei Ren at zmren@sxu.edu.cn). The mitochondrial genome sequences of other aphid species were downloaded from GenBank.

DNA extraction was performed using the Tissue Genomic DNA Extraction Kit from TIANGEN Biotech (Beijing) Co, Ltd. The genomic DNA was sent to Shanghai Majorbio Bio-Pharm Technology Co., Ltd. for library construction and sequenced using the shotgun genome skimming method on an Illumina HiSeq 4000 platform (Zimmer and Wen 2012). The raw data, consisting of 2 × 150 bp paired-end reads and respectively yielding 7.0 and 4.6 GB of data, were filtered using Trimmomatic 3.0 to obtain clean reads (Bolger et al. 2014). De novo assembly was conducted using SPAdes software (Bankevich et al. 2012). The scaffold data were imported into Geneious v10.2.4 for further analysis (Kearse et al. 2012). Using *Chaetogeocia yunlongensis* (Accession No. MF043988) as reference sequences, the two complete mitochondrial genomes of *C. ulmidrupa* were obtained through sequence comparison and alignment. Finally, the physical map of the complete mitochondrial genomes were generated using the Proksee online platform (Grant et al. 2023). The read coverage plots for the complete mitochondrial genomes of *C. ulmidrupa* were presented in Figure S1.

### Phylogenetic analysis

To infer the phylogenetic position of *C. ulmidrupa*, we used a total of 92 mitochondrial genomes from the family Aphididae species with the two species *Adelges laricis* and *A. tsugae* from the family Adelgidae as outgroups. We separately aligned 13 protein-coding genes (PCGs) and two rRNAs of all the aphid sequences. Then, the concatenated alignment matrix was used to construct the molecular phylogenetic tree using the maximum likelihood (ML) method in IQ-TREE v2.1.4 (Nguyen et al. 2015) with the automated substitution model selected by ModelFinder and branch supports quantified exclusively through 1000 UFBoot2 replicates (Hoang et al. 2018) and 1000 SH-aLRT replicates. The tree was then visualized and formatted using the program FigTree v1.4.4 (Rambaut 2018).

## Results

**Figure 2.**
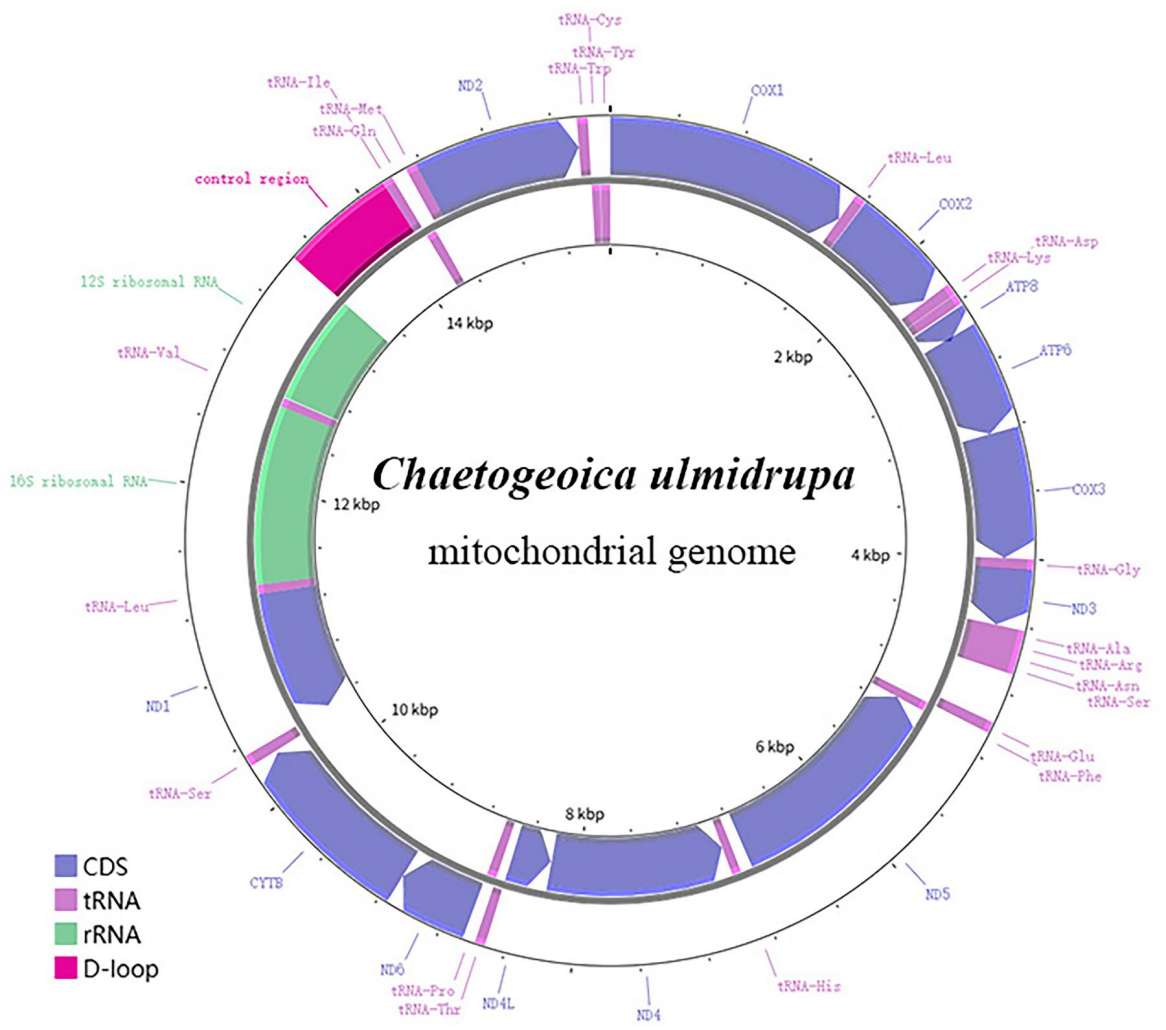
Graphical map of the complete mitochondrial genomes of *Chaetogeoica ulmidrupa.*

These complete mitochondrial genomes of *Chaetogeocia ulmidrupa* were 15,549 and 15,682 bp (Accession number PQ613859 and PV031690) in length (Figure 2), respectively, including 13 PCGs, 22 tRNA genes, two rRNA genes and one control region. The nucleotide composition for the sample Ren_A4512 is 46.3% A, 5.4% G, 9.8% C, and 38.4% T, with an A + T bias of 84.7%. Similarly, the composition for the Ren_A1094 is 46.4% A, 5.4% G, 9.7% C, and 38.4% T, with an A + T bias of 84.8%. The cumulative length of the 13 PCGs is 10,922 bp, with each gene length ranging from 150 bp to 1641 bp. The cumulative length of the tRNA genes is 1469 bp for Ren_A4512 and 1463 bp for Ren_A1094. All the tRNAs have a cloverleaf structure, except for *tRNA-Ser* losing TψC arm. The two rRNA genes, i.e. *rrnL* and *rrnS*, are respectively 1266 bp and 777 bp in length. The control region is located between *rrnS* and *tRNA-Ile,* is 681 bp for Ren_A4512, with the nucleotide composition of 45.8% A, 42.6% T, 6.9% C, 4.7% G, and a GC content of 11.6%. For Ren_A1094, the control region is 723 bp, with the nucleotide composition of 46.1% A, 43.0% T, 6.5% C, 4.4% G, and a GC content of 10.9%. All the PCGs use ATN as start codon, among which the four genes, i.e. *COX3*, *ND4L*, *ND6*, *CYTB*, start with ATG, five genes, i.e. *ND3*, *ATP8*, *COX1*, *COX2*, *ND2*, start with ATA, and the other four genes, i.e. *ND4*, *ND1*, *ND5*, *ATP6*, start with ATT. All the PCGs terminate with TAA except for *COX1* and *ND4* with a single T.

**Figure 3.**
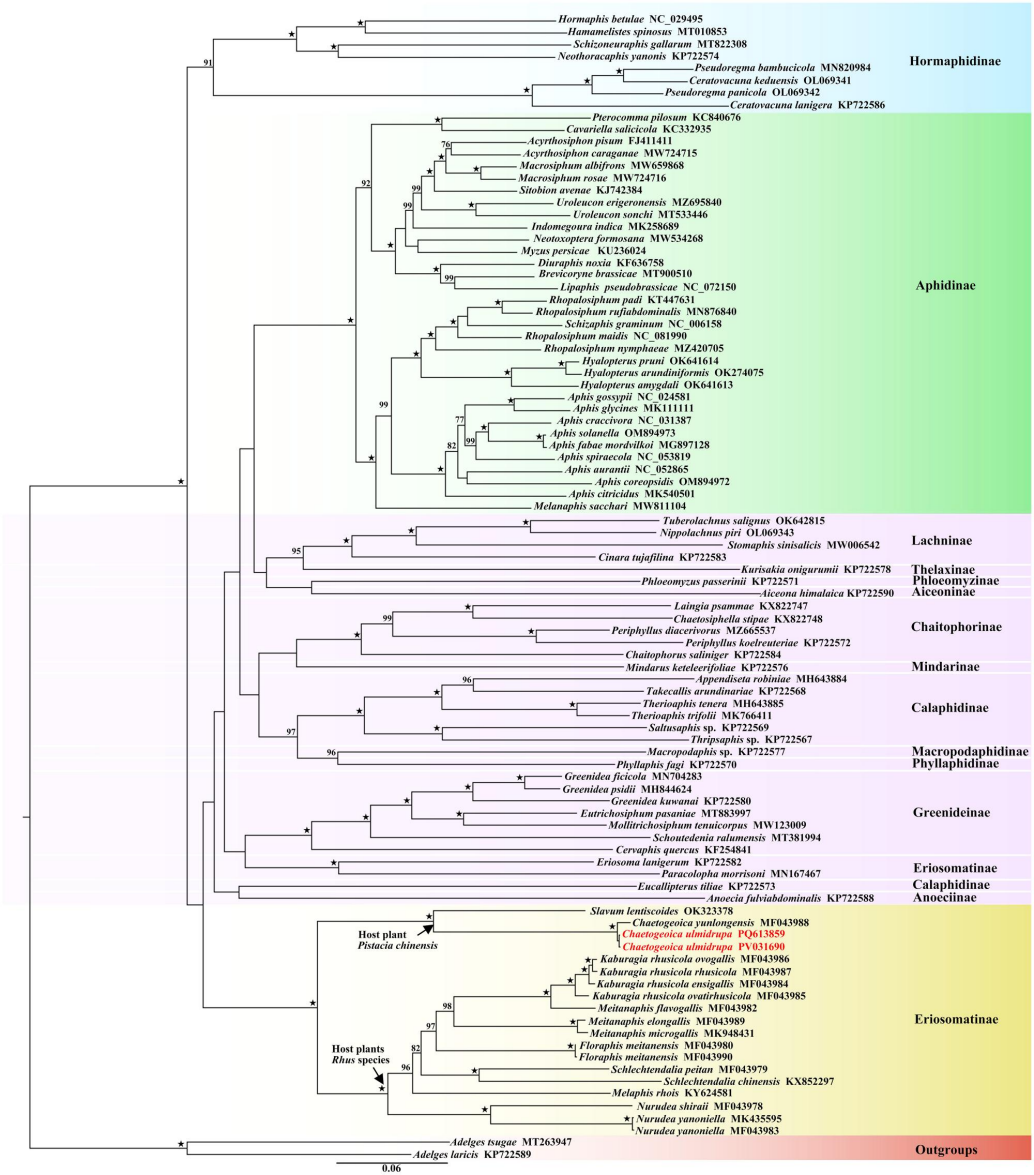
Maximum likelihood phylogenetic tree of aphididae based on 13 PCGs and two rRNA genes. Nodes are labeled with ultrafast bootstrap support values >75%, and ‘★’ represents nodes with 100% BS. The following sequences were used: *Hormaphis betulae* NC_029495 (Li et al. 2017), *Hamamelistes spinosus* MT010853 (Lu et al. 2020), *Schizoneuraphis gallarum* MT822308 (Zhang et al. 2021), *Neothoracaphis yanonis* KP722574 (unpublished), *Pseudoregma bambucicola* MN820984 (Nong et al. 2020), (*Ceratovacuna keduensis* OL069341, *Pseudoregma panicola* OL069342) (Zhang et al. 2022), *Ceratovacuna lanigera* KP722586 (unpublished), (*Pterocomma pilosum* KC840676, *Cavariella salicicola* KC332935) (Wang et al. 2013), (*Acyrthosiphon pisum* FJ411411, *Acyrthosiphon caraganae* MW724715, *Macrosiphum albifrons* MW659868, *Macrosiphum rosae* MW724716) (unpublished), *Sitobion avenae* KJ742384 (Zhang et al. 2016), (*Uroleucon erigeronensis* MZ695840, *Uroleucon sonchi* MT533446) (unpublished), *Indomegoura indica* MK258689 (Hong et al. 2019), (*Neotoxoptera formosana* MW534268, *Myzus persicae* KU236024) (unpublished), *Diuraphis noxia* KF636758 (Zhang et al. 2014), *Brevicoryne brassicae* MT900510 (Li et al. 2021), *Lipaphis pseudobrassicae* NC_072150 (unpublished), *Rhopalosiphum padi* KT447631 (Song et al. 2016), *Rhopalosiphum rufiabdominalis*MN876840 (unpublished), *Schizaphis graminum* NC_006158 (Thao et al. 2004), (*Rhopalosiphum maidis* NC_081990, *Rhopalosiphum nymphaeae* MZ420705) (unpublished), (*Hyalopterus pruni* OK641614, *Hyalopterus arundiniformis* OK274075, *Hyalopterus amygdali* OK641613) (Zhang et al. 2024), *Aphis gossypii* NC_024581 (Zhang et al. 2016), *Aphis glycines* MK111111 (Song et al. 2019), (*Aphis craccivora* NC_031387, *Aphis solanella* OM894973, *Aphis fabae mordvilkoi* MG897128) (unpublished), *Aphis spiraecola* NC_053819 (Du et al. 2019), *Aphis aurantii* NC_052865 (Pu et al. 2020), *Aphis coreopsidis* OM894972 (unpublished), *Aphis citricidus* MK540501 (Wei et al. 2019), *Melanaphis sacchari* MW811104 (unpublished), *Nippolachnus piri* OL069343 (Zhang et al. 2022), *Tuberolachnus salignus* OK642815 (unpublished), *Stomaphis sinisalicis* MW006542 (Zhang et al. 2021), (*Cinara tujafilina* KP722583, *Kurisakia onigurumii* KP722578, *Phloeomyzus passerinii* signoret KP722571, *Aiceona himalaica* KP722590, *Laingia psammae* KX822747, *Chaetosiphella stipae* KX822748, *Periphyllus diacerivorus* MZ665537, *Periphyllus koelreuteriae* KP722572, *Chaitophorus saliniger* KP722584, *Mindarus keteleerifoliae* KP722576, *Appendiseta robiniae* MH643884, *Takecallis arundinariae* KP722568) (unpublished), *Therioaphis tenera* MH643885 (Voronova et al. 2019), *Therioaphis trifolii* MK766411 (Liu et al. 2019), *Saltusaphis* sp. KP722569, *Thripsaphis* sp. KP722567, *Macropodaphis* sp. KP722577, *Phyllaphis fagi* KP722570, (unpublished), *Greenidea ficicola* MN704283 (Liu et al. 2019), *Greenidea psidii* MH844624 (Chen et al. 2019), *Greenidea kuwanai* KP722580 (unpublished), *Eutrichosiphum pasaniae* MT883997 (Li et al. 2020), *Mollitrichosiphum tenuicorpus* MW123009 (Li et al. 2021), *Schoutedenia ralumensis* MT381994 (Chen et al. 2020), *Cervaphis quercus* KF254841 (Wang et al. 2014), *Eriosoma lanigerum* KP722582 (unpublished), *Paracolopha morrisoni* MN167467 (Lee et al. 2019), (*Eucallipterus tiliae* KP722581, *Anoecia fulviabdominalis* KP722588, *Slavum lentiscoides* OK323378, *Chaetogeoica yunlongensis* MF043988, *Kaburagia rhusicola ovogallis* MF043986, *Kaburagia rhusicola rhusicola* MF043987, *Kaburagia rhusicola ensigallis* MF043984, *Kaburagia rhusicola ovatirhusicola* MF043985, *Meitanaphis flavogallis* MF043982, *Meitanaphis elongallis* MF043989) (unpublished), *Meitanaphis microgallis* MK948431 (Liang et al. 2019), (*Floraphis meitanensis* MF043980, *Floraphis meitanensis* MF043990, *Schlechtendalia peitan* MF043979) (unpublished), *Schlechtendalia chinensis* KX852297 (Ren et al. 2016), *Melaphis rhois* KY624581 (Ren and Wen 2017), *Nurudea shiraii* MF043978 (Ren et al. 2017), *Nurudea yanoniella* MK435595 (Ren et al. 2019), (*Nurudea yanoniella* MF043983, *Adelges tsugae* MT263947, *Adelges laricis* KP722589) (unpublished).

The ML phylogenetic tree (Figure 3) supported the monophyly of Hormaphidinae and Aphidinae, while Eriosomatinae and Calaphidinae showed paraphyly. The subfamily Hormaphidinae is located at the base of the tree, and the tribe Fordini (subfamily Eriosomatinae), to which the species *Chaetogeoica ulmidrupa* belongs, is sister to the other 13 subfamilies in the family Aphididae. Additionally, our current species *C. ulmidrupa* is sister to *C. yunlongensis*, and clusters with *Slavum lentiscoides* Mordvilko. These three species all feed on the primary host plant *Pistacia chinensis* and form galls. They are most closely related to the gall aphids that live on the genus *Rhus*, which include six genera and ten species, marked as yellow in Figure 3.

## Discussion and conclusion

In this study, we successfully sequenced and annotated two complete mitochondrial genomes of *Chaetogeoica ulmidrupa*. The gene order and composition are identical to the mitogenome of the same genus species *C. yunlongensis*, which showed the mitochondrial genomic conservation in the same genus.

Our phylogenetic analysis strongly supported the sister relationship between two *C. ulmidrupa* samples, which subsequently clustered with *C. yunlongensis* (BS value of 100%).

The clade consisting of *Chaetogeoica + Slavum lentiscoides* was sister to another one composed of ten species, which use *Rhus* species as their host plants. While, the two genus *Pistacia* and *Rhus* are sister relationship (Yi et al. 2008). Thus, our current results showed a relative phylogenetic relationship between the group of aphids and their host plants, and further to show the effects of the herbivores to insect evolution, i.e. related insect lineages tend to feed on related plants (Lopez-Vaamonde et al. 2003; Sword et al. 2005). Notably, the control region exhibits a low GC content, which may be associated with its high variability and play a role as a transcription initiation site (Dial et al. 2023).

Our study is the first to publish the mitochondrial genome of *C. ulmidrupa*, and the second one in the genus *Chaetogeoica*. Adding more mitochondrial genomes of representative species of the genus *Chaetogeoica* will help clarify the internal evolutionary relationships within the genus. These findings provide molecular data for studying the evolution of Aphididae, and also shed light on the coevolution of aphids and their host plants.

## Supplementary Material

Figure S1.jpg

README_for_Supplemental material.docx

## Data Availability

The genome sequence data that support the findings of this study are available in GenBank of National Center for Biotechnology Information (NCBI, https://www.ncbi.nlm.nih.gov/) under the accession numbers PQ613859 and PV031690. The associated BioProject, SRA, and BioSample numbers are as follows: PRJNA1193212 and PRJNA1208921; SRR31578817 and SRR32023171; SAMN45133405 and SAMN46192941, respectively.
