# Supplementary figures and images for: Complete mitochondrial genome and phylogenetic position of the gall aphid Chaetogeoica ulmidrupa (Hemiptera: Aphididae)
Source: Mitochondrial DNA B Resour. 2026 Mar 5;11(4):498–503. doi: 10.1080/23802359.2026.2638669 (PMC12964470; doi:10.1080/23802359.2026.2638669)

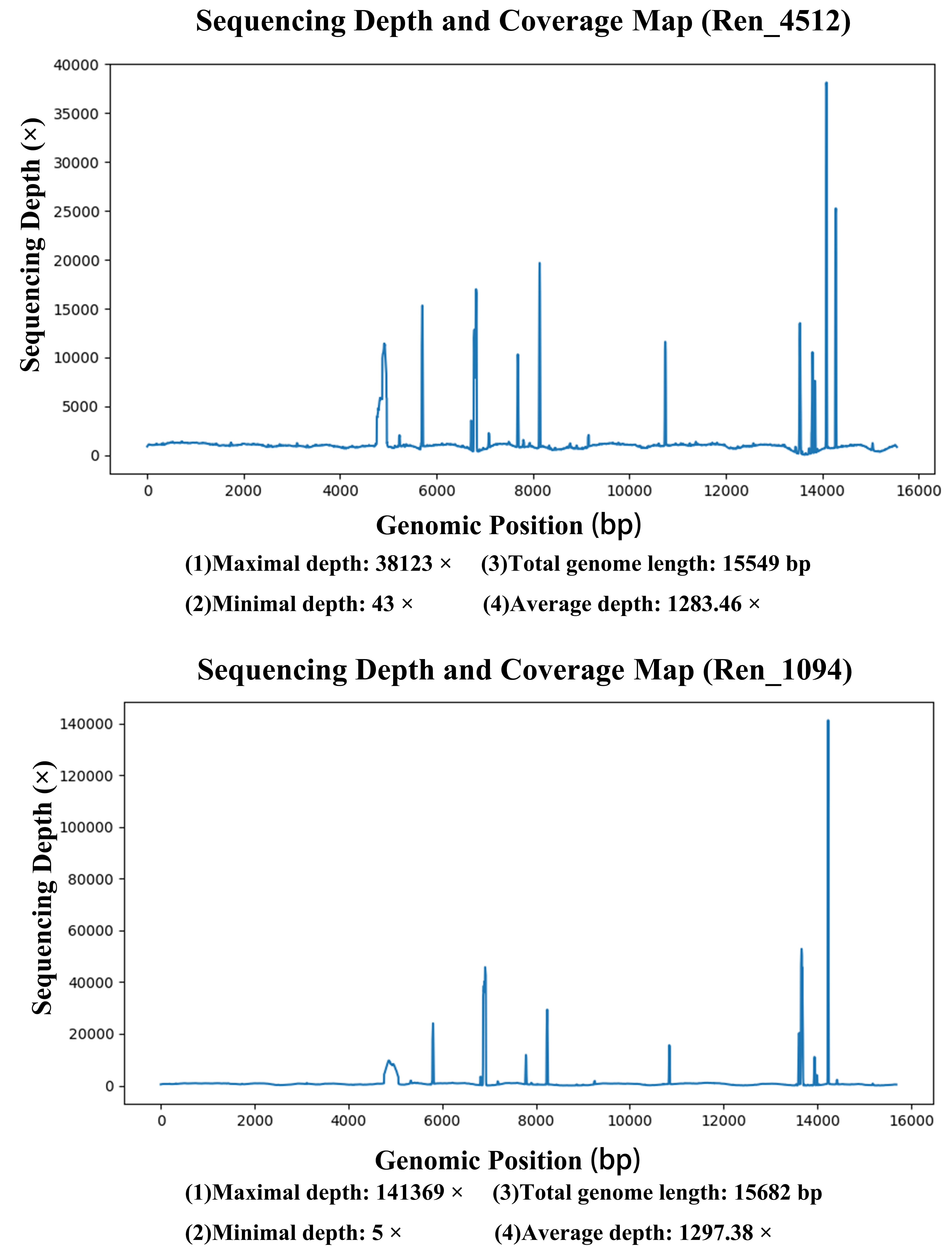

Supplement: Figure S1.jpg [file TMDN_A_2638669_SM9065.jpg]
