# Supplementary material for: Complete mitochondrial genome and phylogenetic position of the gall aphid Chaetogeoica ulmidrupa (Hemiptera: Aphididae)
Source: Mitochondrial DNA B Resour. 2026 Mar 5;11(4):498–503. doi: 10.1080/23802359.2026.2638669 (PMC12964470; doi:10.1080/23802359.2026.2638669)
Supplement: README_for_Supplemental material.docx [file TMDN_A_2638669_SM9064.docx]

**Supporting data:**

**Figure S1.** Coverage with sequencing depth of *C. ulmidrupa* mitogenome, Ren_4512 and Ren_1094.
